# Cecal Lipoma Presenting as Acute Intestinal Obstruction in an Elderly Woman: A Case Report

**DOI:** 10.1155/2013/926514

**Published:** 2013-06-20

**Authors:** Miltiades Kastanakis, Dimitrios Anyfantakis, Emmanouil K. Symvoulakis, Nikolaos Katsougris, Alexandros Papadomichelakis, Ioannis Kokkinos, George Petrakis, Emmanouil Bobolakis

**Affiliations:** ^1^First Department of Surgery, Saint George General Hospital of Chania, 73100 Crete, Greece; ^2^Primary Health Care Centre of Kissamos, Chania, 73400 Crete, Greece; ^3^Private Family Practice Unit in Heraklion, 74100 Crete, Greece

## Abstract

Colonic lipomas are rare nonepithelial tumors that are usually detected incidentally during surgery or colonoscopy. Although lipomas generally remain asymptomatic, when they exceed 2 cm of diameter they may cause abdominal pain, obstruction, or intussusception. Here we present a case of an elderly woman referred by her general practitioner to a general hospital of Crete because of acute abdominal pain along with signs of intestinal obstruction and a positive history of appetite loss. Abdominal computed tomography was performed. To marginalise the risk of malignancy, a right hemicolectomy was performed. Histopathological examination of the resected specimen confirmed the diagnosis of cecal lipoma.

## 1. Introduction

Colonic lipomas are rare nonepithelial tumors of the large intestine which are usually detected incidentally during surgery or endoscopy [1]. Their incidence ranges from 0.2% to 4.4% [2]. Those with a diameter smaller than 2 cm usually remain asymptomatic [1]. Larger intestinal lipomas may cause symptoms of intestinal obstruction, intussusception, rectal bleeding, or diarrhea [1, 3]. Preoperative diagnosis is often challenging [4]. We describe a case of massive cecal lipoma in an elderly female presenting with signs of acute intestinal obstruction.

## 2. Case Report

**Figure 1:**
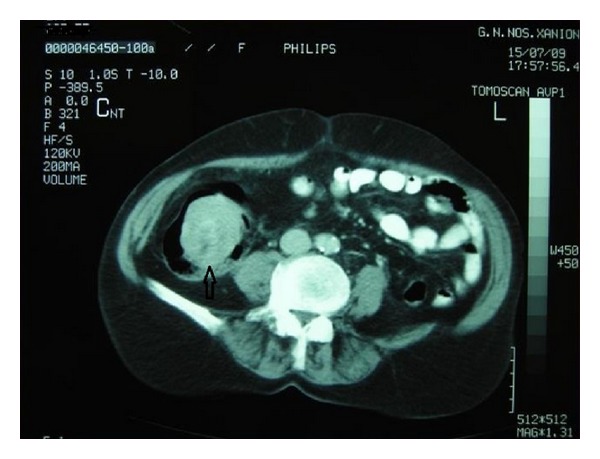
Contrast-enhanced abdominal CT revealing the presence of a mass at the cecum level (arrow).

A 68-year-old Caucasian Greek woman was referred to the Surgery Department of the Saint George General Hospital of Chania, Crete by her general practitioner due to a history of intermittent right abdominal pain, meteorism, and constipation lasting for the last 3 days. She also reported loss of appetite during the last month. Her past medical history was unremarkable. The patient did not report any history of anemia or episodes of diarrhea or rectal bleeding. On abdominal examination, palpation revealed slight abdominal tenderness on the right lower quadrant and auscultation increased bowel sounds. Laboratory workup including complete blood count, renal and liver function tests, and blood coagulation was within normal limits except of an elevated level of C-reactive protein (1.7 mg/dL, range: 0–0.5 mg/dL). Contrast-enhanced abdominal CT disclosed the presence of a well defined, ovoid mass of 5 × 7 cm in size protruding into the lumen of the cecum (Figure 1). Due to the suspicion of malignancy, the patient was operated with the priority of an emergency case, and a right hemicolectomy was performed. The resected specimen was a homogeneous tumor rising from the submucosa. Histopathological examination of the resected specimen confirmed the diagnosis of a submucosal cecal lipoma. The postoperative course was uneventful, and the patient was discharged home on the 15th day postoperatively.

## 3. Discussion

Lipomas of the gastrointestinal tract were first described by Bauer in 1757 [5]. A preponderance for the female gender between 4th and 7th decade of life has been reported [3, 6]. They are more often located in the right hemicolon. Cecum lipomas account for approximately 20% of the colonic lipomas. These are rare neoplasms of mesenchymal origin that are presented as a polypoid tumor that protrude into the intestinal lumen [1, 3]. In the majority of cases, they arise from the submucosa [1]. Rarely, in 10% of the cases they extend to subserosa [7]. Chronic inflammation of the cecum may have a role in the etiopathogenesis through a mechanism of increased intestinal motility that causes consequently detachment of mucosa [7]. Their dimension extends from 2 cm to 30 cm [6]. They are usually solitary, but they can be also multiple [8].

Only a quarter of the colonic lipomas become symptomatic [6]. Abdominal pain, obstruction, rectal bleeding, and intussusception are commonly reported when the condition becomes symptomatic [1]. Abdominal pain ranges from intermittent colicky pain to severe acute abdominal pain. Duration of symptoms can range from days to years. Lipomas greater than 2 cm are symptomatic in many cases and may cause luminal obstruction of the enlarged mass [6]. 

 Abdominal CT represents a valuable noninvasive imaging modality for the diagnosis of colonic lipomas [1]. Barium enema may disclose an ovoid filling defect with well delineated limits [9]. Colonoscopy is the suggested procedure when a diagnosis of colonic cancer is suspected [1]. Submucosal lipoma is visualized as a smooth, spheric polypoid mass covered by mucosa [2, 3]. Endoscopic characteristics include the “tending sign” (grasp the overlying mucosa) and “cushion sign” (flattening of the lipomas form) [7]. In some cases, endoscopy may reveal ulcerative lesions of the polypoid mass leading to a presumption of malignancy [9].

Management of colonic lipomas includes endoscopy and surgery. Polypectomy through endoscopy is recommended for lipomas that are considered of low complication risk [1]. These are lipomas with a diameter less than 2 cm or pedunculated lipomas with thin tail. Surgery represents the standard therapeutic option for lipomas with diameter greater than 2 cm [6]. On the basis of the lipomas size and location, surgical management includes resection, colotomy with local excision, hemicolectomy, or subtotal colectomy [1]. Laparoscopic resection under colonoscopic guidance of symptomatic lipomas of the colon has been also reported [9].

Colonic lipomas pose a preoperative diagnostic difficulty, since they may be asymptomatic for years or may be misdiagnosed as malignant tumors [7]. Atypical etiologies of intestinal obstruction should be considered in patients with persistent intermittent abdominal discomfort. Abdominal tumor conditions, even though benign, may be presented as cases of emergency care. In this case report, the option of an immediate surgical approach by performing a right hemicolectomy was suggested since intestinal obstruction signs were present and malignancy was likely to occur.
